# Molecular Characterization of PGC-1β (PPAR Gamma Coactivator 1β) and its Roles in Mitochondrial Biogenesis in Blunt Snout Bream (*Megalobrama amblycephala*)

**DOI:** 10.3390/ijms21061935

**Published:** 2020-03-12

**Authors:** Kangle Lu, Tomas Policar, Xiaojun Song, Samad Rahimnejad

**Affiliations:** 1 Laboratory of Fish Nutrition and Physiology, Fisheries College, Jimei University, Xiamen 361021, China; 201561000041@jmu.edu.cn; 2 Faculty of Fisheries and Protection of Waters, University of South Bohemia in Ceske Budejovice, South Bohemian Research Center of Aquaculture and Biodiversity of Hydrocenoses, Zatisi 728/ II, 389 25 Vodnany, Czech Republic; policar@frov.jcu.cz; 3 College of Marine Science and Engineering, Qingdao Agricultural University, Qingdao 266109, China

**Keywords:** PPAR, mitochondrial biogenesis, NRF-1, fish model

## Abstract

This study aimed at achieving the molecular characterization of peroxisome proliferator-activated receptor-gamma coactivator 1β (PGC-1β) and exploring its modulatory roles in mitochondria biogenesis in blunt snout bream (*Megalobrama amblycephala*). A full-length cDNA of *PGC-1β* was cloned from liver which covered 3110 bp encoding 859 amino acids. The conserved motifs of PGC-1β family proteins were gained by MEME software, and the phylogenetic analyses showed motif loss and rearrangement of *PGC-1β* in fish. The function of PGC-1β was evaluated through overexpression and knockdown of PGC-1β in primary hepatocytes of blunt snout bream. We observed overexpression of *PGC-1β* along with enhanced mitochondrial transcription factor A (*TFAM*) expression and mtDNA copies in hepatocytes, and its knockdown led to slightly reduced *NRF1* expression. However, knockdown of PGC-1β did not significantly influence *TFAM* expression or mtDNA copies. The alterations in mitochondria biogenesis were assessed following high-fat intake, and the results showed that it induces downregulation of *PGC-1β*. Furthermore, significant decreases in mitochondrial respiratory chain activities and mitochondria biogenesis were observed by high-fat intake. Our findings demonstrated that overexpression of PGC-1β induces the enhancement of TFAM expression and mtDNA amount but not NRF-1. Therefore, it could be concluded that PGC-1β is involved in mitochondrial biogenesis in blunt snout bream but not through PGC-1β/NRF-1 pathway.

## 1. Introduction

The peroxisome proliferator-activated receptor-gamma coactivator 1 (PGC-1) family including PGC-1α, PGC-1β, and PRC are critical transcriptional coactivators regulating mitochondrial biogenesis and energy metabolism in mammals [1]. PGC-1α was firstly characterized as a stimulator of thermogenin in mice [2], and later PGC-1β and PRC were discovered by searching for PGC1α homologues through database [3]. Among these three family members, PGC-1α has been studied mostly and is known to exert an array of well-defined roles in mammals [4]. Although the three PGC1 proteins have some common features, they differ in some distinct functions. In mammals, activated PGC-1α mostly induces the transcription of several genes involved in mitochondrial biogenesis including nuclear respiratory factor 1 (NRF-1) and mitochondrial transcription factor A (TFAM). Despite the establishment of PGC-1α/NRF-1/TFAM pathway in mammals, this axis has been rarely studied in lower vertebrates including fish [5,6]. Interestingly, it has been reported by several authors that the changes of PGC-1α in the temperature- and nutrition-induced mitochondrial remodeling in fish are inconsistent with its central role as a master regulator [6,7]. Furthermore, it has been pointed out that the PGC-1α/NRF-1 pathway is probably disrupted in fish because of fish PGC1α orthologs insertion in the region severing as NRF-1 binding domain in mammals [8,9].

Usually, mammalian PGC-1α and PGC-1β share several roles such as engagement in mitochondrial biogenesis and adaptation to fasting, nutrition, and exercise [10,11,12]. It is assumed that PGC-1β plays more crucial roles than PGC-1α in promoting fuel oxidation in muscle and liver [13]. In mammals’ skeletal muscle, PGC-1β could be stimulated by medicines intake, which can subsequently lead to enhanced mitochondria content, whereas this is not true about PGC-1α or PRC [14]. It is speculated that in fish PGC-1α has lost the ability to interact with NRF-1 as a result of structural changes, thus, instead, it is assumed that PGC-1β plays the central role in controlling mitochondrial biogenesis in fish [8,15].

The objectives of the present research were to (1) achieve the molecular characterization of PGC-1β and (2) to assess the role of *PGC-1β* in the regulation of mitochondria biogenesis and bioenergetics in blunt snout bream (*Megalobrama amblycephala*), which is a popular candidate for aquaculture industry in China due to its rapid growth, tender flesh, and high disease resistance.

## 2. Results

### 2.1. Identification and Characterization of PGC-1β

The full cDNA of *PGC-1β* gene from *M. amblycephala* was 3110 bp (GenBank accession number: MH791035). In addition to a 440 bp 3′-untranslated region and 90 bp 5′-untranslated region, it had an ORF of 2580 bp, encoding an 859 amino acid polypeptide, and contained a canonical polyadenylation signal (AATAA) (Supplemental Figure S1). Given the PGC-1 family members contain a conserved RNA recognition motif domain in the C-terminal part, using SMART software, we found that the *M. amblycephala* PGC-1β protein also contains an RRM domain in C-terminal.

The results of multisequence analysis showed that fish PGC-1β proteins have three conserved motifs including LXXLL, TPPTTPP, and DHDYC motifs with mammals PGC-1β proteins (Supplemental Figures S2 and S3). However, the putative phosphorylation sites of PGC-1β proteins are different between fish and mammals (Table 1).

### 2.2. Phylogenetic Analyses

**Figure 1:**
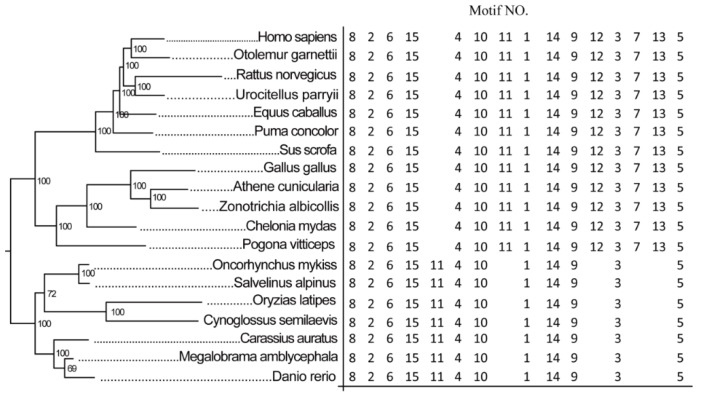
Phylogenetic and MEME analysis of peroxisome proliferator-activated receptor-gamma coactivator-1β (PGC-1β) family proteins between different animals. On the left is the phylogenetic tree of PGC-1β family proteins in different animals using Bayesian inference (BI), the right panel shows the conserved motif form of PGC-1β proteins in corresponding animals by MEME software. The topology of the BI tree revealed the evolutionary relationships of PGC-1β with the precision of topology supported by a high bootstrap value. The composition of conserved motif showed motif loss and rearrangement between fish and mammals.

**Figure 2:**
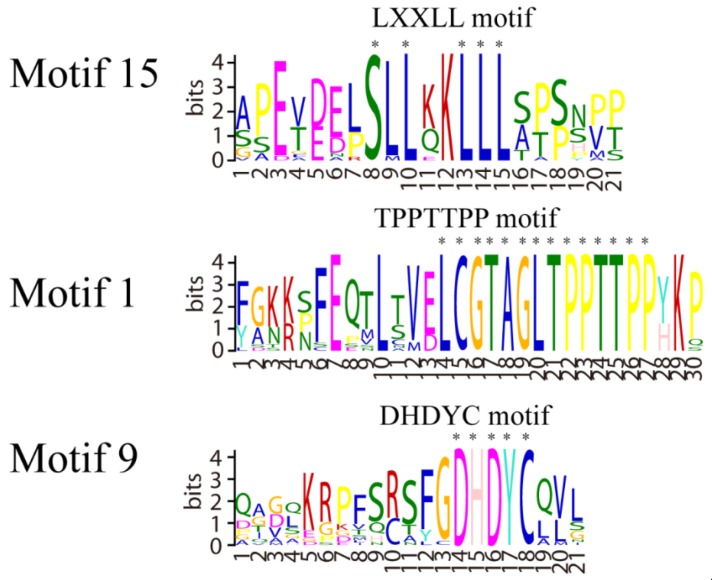
The conserved motif of PGC-1β family proteins in fish and mammals. MEME results showed that there are three conserved motifs (LXXLL, TPPTTPP, and DHDYC) in fish and mammals. X means any amino acid residue and the letter size indicates different conservation. The functions of these domains are as following: LXXLL motif plays a crucial role in the interactions between the coactivators and liganded nuclear receptors, TPPTTPP motif can bind to PPARδ or phosphorylation, DHDYC motif is the putative binding site of host cell factor 1 (HCF1).

The evolutionary relationships of PGC-1β proteins were revealed by the topology of a phylogenetic tree supported by high bootstrap values (Figure 1). PGC-1β of *M. amblycephala* clustered with *Danio rerio* PGC-1β protein. The conserved motifs of PGC-1β family proteins were gained by MEME software, and the composition of conserved motif showed motif loss and rearrangement between fish and mammals (Figure 2). Compared to mammals, the fish PGC-1β lacked Motif 7, Motif 12, and Motif 13, located at N-terminal which is an important part of the RRM domain. Therefore, the motif loss in N-terminal can affect the function of RRM domain. Furthermore, the location of Motif 11 was rearranged in fish PGC-1β as compared with mammals.

We used PAML4.4 to analyze the changes in selective pressure and the results are presented in Supplemental Table S3. The findings revealed that *PGC-1β* genes underwent very strong purifying selection (ω = 0.19). Nine positive selective sites were detected by M7 vs. M8 model. To explore the functional and evolutionary differences in *PGC-1β* genes between fish and other vertebrates, the specific positive selective sites in fish were detected by branch-site model of PAML software package. Our results showed that there were four positive selective sites (*P* < 0.05) detected by Bayes empirical Bayes (BEB) methods of branch-site model.

### 2.3. Expression of PGC-1β in Tissues

**Figure 3:**
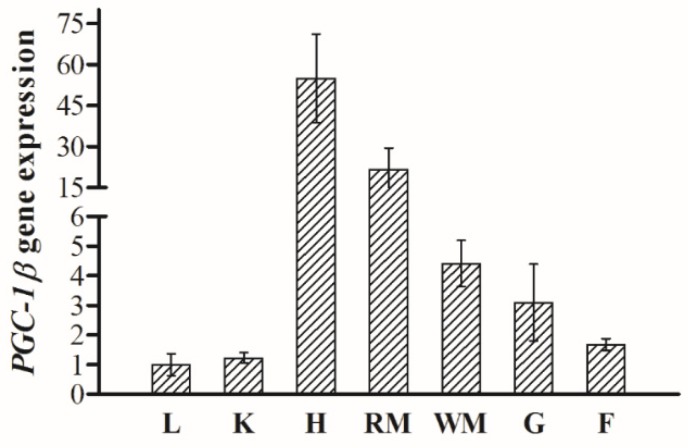
Expression of PGC-1β gene in different tissues of blunt snout bream (*M. amblycephala*) (N = 6) (L, liver; K, kidney; F, fat tissue; RM, red muscle; WM, white muscle; H., heart; G, gill).

*PGC-1β* gene was ubiquitously expressed with varying levels in all the tested tissues. As shown in Figure 3, the highest expression of *PGC-1β* was observed in heart followed by red muscle, white muscle, gill, fat tissue, kidney, and liver, respectively.

### 2.4. Mitochondrial Biogenesis after Overexpression and Knock Down of PGC-1β

**Figure 4:**
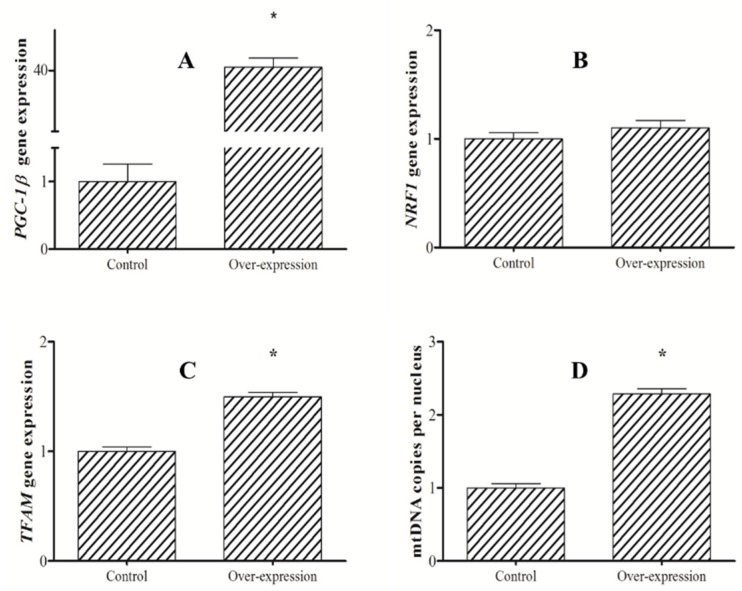
Expression of PGC-1β (**A**), NRF1(**B**) and mitochondrial transcription factor A (TFAM) (**C**); and mtDNA (**D**) copies after overexpression of PGC-1β in hepatocytes of blunt snout bream (*M. amblycephala*). The asterisk indicates significant difference (*P* < 0.05, Student’s t-test).

To assess the *PGC-1β* roles directly, we knocked down and upregulated PGC-1*β* expression in primary hepatocytes of blunt snout bream and explored the effects on mitochondrial biogenesis. Overexpression of *PGC-1β* occurred along with a significant elevation in *TFAM* expression and mtDNA copies. However, *PGC-1β* overexpression did not enhance *NRF-1* expression (Figure 4).

**Figure 5:**
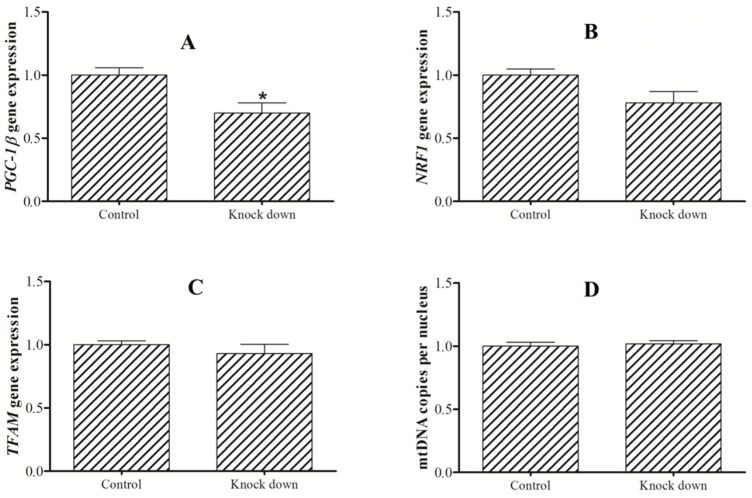
Expression of PGC-1β (**A**), NRF1(**B**) and TFAM(**C**), and mtDNA(**D**) copies after knockdown of PGC-1β in hepatocytes of blunt snout bream (*M. amblycephala*). The asterisk indicates significant difference (*P* < 0.05, Student’s t-test).

Furthermore, knockdown of *PGC-1β* did not significantly influence *TFAM* expression and mtDNA copies. However, slightly lower *NRF-1* expression was detected in the knockdown group, although the difference was not statistically significant (*P* = 0.055) (Figure 5).

### 2.5. Mitochondrial Biogenesis and Bioenergetics after High-Fat Intake

**Figure 6:**
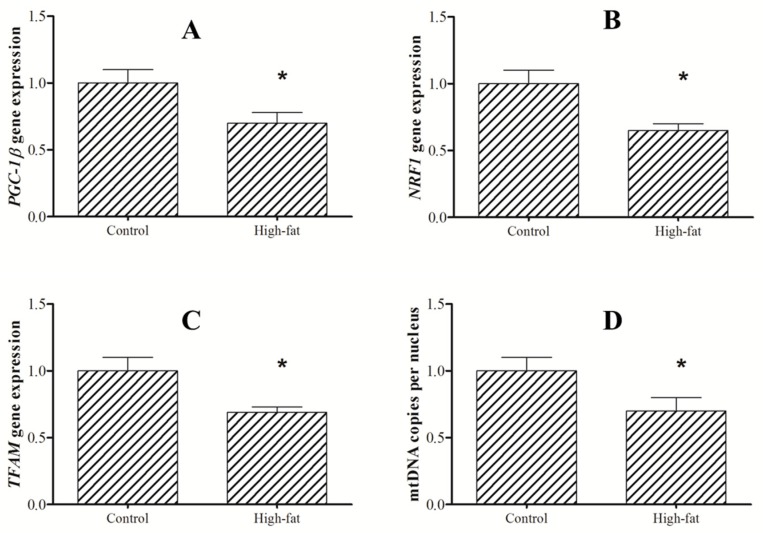
Expression of PGC-1β (**A**), NRF-1(**B**) and TFAM(**C**), and mtDNA(**D**) copies in liver of blunt snout bream (*M. amblycephala*) fed control (5% fat) or high-fat diet (HFD, 15% fat) for 10 weeks. The asterisk indicates significant difference (*P* < 0.05, Student’s t-test).

Dietary fat content influenced biogenesis and bioenergetics identified by the changes in mtDNA copies and mitochondrial enzymes activity. Compared to the control group, there was a significant decrease in the Complex I and CS activities of mitochondrial respiratory chain in response to high-fat diet (Table 2). Similarly, lower expression of *NRF1* and *TFAM*, and mtDNA copies were detected in the high-fat group. Moreover, high-fat intake could downregulate the expression of *PGC-1β* (Figure 6).

## 3. Discussion and Conclusions

The PGC-1 family has drawn widespread attention over the past decades as they are engaged in master regulation of energy metabolism and mitochondrial function [16]. Although PGC-1 family orthologs have been recognized in fish, less attention has been paid to their molecular characterization and functional analysis, particularly those of PGC-1β. The analysis of PGC-1β cloned from liver of *M. amblycephala,* in the current study, suggested that fish PGC-1β proteins have conserved motif with mammals, but the putative phosphorylation sites of PGC-1β proteins are different between fish and mammals. Furthermore, the results of multisequence analysis showed that fish PGC-1β proteins have three conserved motifs such as LXXLL, TPPTTPP, and DHDYC with PGC-1 family proteins in mammals. The LXXLL motif plays a crucial role in the interactions between the coactivators and liganded nuclear receptors including thyroid receptor, hepatocyte nuclear factor, PPARα, and retinoid X receptors [17]. This finding provides evidence that fish PGC-1β has the nuclear hormone receptor motif. The DHDYC motif is the putative binding site of host cell factor 1 (HCF1), a major chromatin component which mediates the association between PGC-1 and NRF-1 [18]. The TPPTTPP site is the perfect conservation throughout vertebrates indicating its important role. According to the literature, TPPTTPP functions through binding to PPARδ or phosphorylation by P38MAPK [19]. Moreover, it has been shown that PGC-1β of *M. amblycephala* has much more putative phosphorylation sites than those of the zebrafish and human. Phosphorylation is very important for this family due to the existence of an autoregulatory loop through which more active PGC-1α, PGC-1β, and PRC stimulate their own transcription in fish and mammals [8]. The different putative phosphorylation sites of *M. amblycephala* PGC-1β can lead to species-specific functional differences.

It has been reported that tissue expression patterns of PGC-1β in rodents and humans is conserved [17]. In mice, PGC-1β is expressed at high levels in heart, brown adipose tissue, and brain, and it can be detected at low levels in the other tissues [17]. PGC-1β is enriched in tissues with abundant mitochondria content such as heart, skeletal muscle, and brown fat [17]. Similarly, in this study, the highest expression of PGC-1β was observed in heart and red muscle. In addition, among the tested tissues in the current study, the highest quantity of mitochondria was found in heart and red muscle, owing to their high metabolic rate. Moreover, PGC-1β was highly expressed in fat tissue probably indicating its role in differentiation and development of adipocytes [20]. This is consistent with previous findings in human studies where PGC-1β mRNA level in white adipose tissue was higher than in the skeletal muscle and lower than in the liver [20,21]. Surprisingly, despite of the fact that liver is the main tissue for fatty acid oxidation in fish, in this study, expression of PGC-1β in liver was even lower than that of fat tissue. This could be due to the fact that mitochondria play an important role in adipogenesis, and it has been reported that the number of mitochondria remarkably increases during adipocyte differentiation under transmission electron microscopy [22].

PGC-1 family is well known for regulation of mitochondrial biogenesis and fatty acids metabolism in mammals and birds [1]. However, as pointed out by several authors, fish PGC-1α does not play a role in mitochondrial biogenesis because of the disruption in the PGC-1α/NRF1 pathway [5,8]. LeMoine et al. [6] suggested that PGC-1α lacks the potential binding site for NRF-1 due to successive serine- and glutamine-rich insertions. Furthermore, immunocopurification revealed that recombinant NRF-1 protein binds PGC-1α in mammals but not in fish [8]. Moreover, there are several reports that PGC-1α mRNA was not responsive to swimming, cold acclimatization, and feed composition [7,23]. Accordingly, some researchers speculate that PGC-1β plays the central role in controlling mitochondrial biogenesis in fish [8,15].

As PGC-1β can play greater roles in mitochondrial biogenesis in fish, the overexpression and knockdown approaches were implemented to study its characterization. The results showed that the overexpression of *PGC-1β* induces upregulation of *TFAM* and mtDNA copies. This agrees with the results of studies on some mammalian cells, where upregulation of *PGC-1β* often resulted in a robust increase in mtDNA copies [24]. This observation confirms the regulatory effect of PGC-1β on *TFAM* expression and mitochondrial biogenesis in fish. TFAM is a primary factor controlling the mtDNA copy number in cells, and the increased concentration of intra-mitochondrial TFAM was sufficient to stimulate mitochondrial transcription [25]. It has been reported that PGC-1 coactivators upregulate the expression of TFAM and drive an increase in replication and expression of mtDNA. Moreover, PGC-1 coactivators regulate TFAM mainly via NRF-1 and estrogen-related receptor α (ERRα) in mammals [25]. However, in the present study, the expression of *NRF-1* was not affected after overexpression of *PGC-1β*. In general, PGC-1 coactivators and NRF-1 bind together and form a heterodimer for regulating mitochondrial function [1]. The binding of NRF-1 and PGC-1 coactivators enhances the transcription of NRF-1. In mammals, PGC-1 coactivators can profoundly induce the expression of *NRF-1* [26]. Mammal NRF-1 can bind PGC-1α at the ill-defined region spanning amino acids 180 to 403 [1]. Whereas, the interaction of NRF-1 and PGC-1β dictated by the middle HBM domain (351 to 750 amino acids) of PGC-1β is confirmed in human and rat [27]. Our results, however, show that PGC-1β does not induce the expression of NRF-1 in fish. PGC-1β exhibits different functions from PGC-1α, as a recent report confirmed the direct interaction of ERRα [28]. Mammal ERRa by siRNA ablated the function of PGC-1β for the oxidative phosphorylation and mitochondrial biogenesis [27]. According to our results, we assume that fish PGC-1β regulation of mitochondrial biogenesis is mediated through PGC-1β/ERRα and through the PGC-1β/NRF-1 pathway. Likewise, a recent zebrafish study suggested that PGC-1β regulates the expressions of important mitochondrial genes through ERRα [5].

The knockdown of PGC-1β did not influence the expression of NRF-1, TFAM, and mtDNA. We assume that this is due to possible compensatory effects exerted by PGC-1 coactivators. It has been demonstrated that cells deficient in either PGC-1α or PGC-1β coactivators do not show decreased mitochondrial biogenesis and respiration, while the increase in mitochondrial number and function was totally abolished when both PGC-1α and PGC-1β were deficient [12]. The PGC-1 coactivators play an absolutely essential but complementary function in mitochondrial biogenesis [12]. In addition, as PGC-1β expression is only 25% reduced, the other 75% could still regulate mitochondrial biogenesis.

Mitochondrion as an integral component of cellular energy metabolism is highly responsive to a variety of physiological changes [29,30]. Cold exposure can increase cellular energy demand, and then trigger mitochondrial biogenesis [6]. Additionally, there are some chemical or nutritional inducers of mitochondrial biogenesis, such as resveratrol and fat-rich diets [31]. In this study, we explored the putative regulatory role of PGC-1β and its roles in the metabolic adaptations induced by the diet composition. Several dietary components such as resveratrol [32] and fat content [33,34] influence the mitochondrial biogenesis. In this study, a significant reduction in mtDNA copy number was found in the high-fat group which is consistent with the results of PGC-1β, NRF-1, and TFAM expression. The downregulation of *PGC-1β* in the high-fat group implied the correlation between *PGC-1β* expression and mitochondrial biogenesis. However, it is hard to explain the alteration of NRF-1 expression, as we suppose that regulation of mitochondrial biogenesis by PGC-1β is not mediated through the PGC-1β/NRF-1 pathway. Moreover, PGC-1 promotes metabolic reprogramming by activating enzymes involved in mitochondrial fuel catabolism [35]. It has been reported that high-fat intake often impairs mitochondrial bioenergetics and induces obesity, diabetes, and fatty liver [36,37]. In addition, in this study, fish fed high-fat diets exhibited signs of hepatic steatosis. Analysis of the mitochondrion-related enzymes activity in this study revealed significant reductions in the Complex I and CS activities of mitochondrial respiratory chain in response to high-fat intake, and the alteration of mitochondrial bioenergetics seems to be as a result of PGC-1β downregulation. It should be pointed out that dietary fish oil can also stimulate metabolism independently from PGC-1β, however, this could not be the case in this study as both control and high-fat diets contain fish oil varying only in lipid level.

To conclude, our findings demonstrated that overexpression of PGC-1β induces increased TFAM expression and mtDNA amount but did not affect the NRF-1 expression. Therefore, we suggest that PGC-1β is involved in mitochondrial biogenesis in fish but not through the PGC-1β/NRF-1 pathway.

## 4. Materials and Methods

### 4.1. Experimental Fish and Sample Collection

The experimental fish were transported from a private hatchery (Guangzhou, China) to the aquaculture facility of Jimei University. The fish were cultured in a recirculating aquaculture system (RAS). During one week of the adaptation period, the range of water quality parameters including water temperature, dissolved oxygen, and pH were 25 to 27 °C, 5.0 to 6.0 mg l^−1^, and 7.2 to 7.6, respectively. At the end of the acclimatization, tissue samples including liver, kidney, fat tissue, red muscle, white muscle, heart, and gill were collected after anesthetization with 0.01% MS-222 (tricaine methanesulfonate, Sigma) for gene cloning. All the fish were treated in compliance with the ARRIVE guidelines and the principles of the Basel Declaration and Recommendations of Animal Research Institute Committee guidelines, Jimei University, China.

### 4.2. cDNA Cloning of PGC-1β

Firstly, total RNA from liver was extracted using a commercial kit (RNAiso Plus, Takara Co. Ltd., Japan). Subsequently, the quantity and purity of isolated RNA were determined by absorbance measurements at 260 and 280 nm, and electrophoresis was used for testing its integrity. To obtain the full cDNA of *PGC-1β*, the rapid amplification of cDNA ends (RACE) was used, as in our previous study (Song et al., 2019). The primers designed for 5′ and 3′ RACE are presented in Supplemental Table S1. The 5′ RACE System of Invitrogen was used for rapid amplification of the 5′ end. Briefly, to obtain the first strand cDNA, a 2 mg sample RNA with PGC1β-5-R was used. Following RNase treatment, an Oligo (dC) at the 5′ end was supplemented using terminal deoxynucleotidyl transferase. The resultant product served as a template for the initial PCR amplification at 94 °C for 2 min and 30 amplification cycles at 94 °C for 30 s, 55 °C for 30 s, 72 °C for 60 s, and 72 °C for 7 min. Thereafter, the first PCR product served as a template for the nested PCR. The product of nested PCR was eluted from 1% agarose gel and sent to Shanghai Sangon Biotech Service Co. Ltd. (Shanghai, China) for sequencing. The 3′ end rapid amplification was done using the 3′-full RACE Core Set (TaKaRa, Dalian, China) based on the manufacturer’s instructions. Finally, after the first and nested PCRs (consisted of 25 cycles of 30 s at 94 °C, 30 s at 65 °C, and 1 min at 68 °C), a PCR purification kit (QIAGEN, United States) was used to elute the PCR product from 1.0% agarose gel, and sequenced.

### 4.3. Alignment, Phylogenetic, and Codon-Based Sequence Analysis

The homology in sequences of PGC-1β proteins of different animals was assayed through multiple sequence alignment using MUSCLE with default parameters. Gene structure and position of motifs were checked using data from Entrez genes, and domain conservation was predicted by SMART. Conserved motifs were analyzed online using a MEME system Version 5.0.5. Phylogenetic analyses were conducted using MrBayes 3.2 [38]. The putative phosphorylation sites of PGC-1β proteins in fish and mammals were predicted by NetPhos 3.1 Server. Here, a codon-substitution model implemented in the CODEML program in the PAML4.4 software package was used to analyze changes in selective pressure, as in our previous study [9].

### 4.4. Overexpression of PGC-1β

The procedures are as follows:

**Plasmid construction** The CDS of *PGC-1β* was ligated to the pcDNA3.1+ vector (Invitrogen, CA, USA) to construct the plasmids pcDNA3.1+-PGC-1β. And the lentiviral vector of LV2-PGC-1β was constructed according to the manufacturer’s instructions (GenePharma, Shanghai, China). All the plasmids used in this study were verified by DNA sequencing at Sangon (Shanghai, China).

**Packaging and concentrating of lentivirus** 293T cells were co-transfected with pGag/Pol, pRev, pVSV-G, and LV2-PGC-1β vectors, and the virus was packaged, then, the virus stock was collected and concentrated by ultrafiltration.

**Lentivirus infection of primary hepatocytes** Primary hepatocytes of blunt snout bream were isolated and cultured as our previous study [9]. Cells at 24 h after seeding were subjected to the infection of lentiviruses with the presence of 6 μg/mL polybrene. For each infection, a total of 1.0 × 10^8^ lenti-virions were added and incubated for 4 h. Thereafter, the medium containing unabsorbed lenti-virions was replaced by medium. The cells were harvested for measuring the expression of PGC1β at 72 h postinfection.

### 4.5. Knockdown of PGC-1β

Hepatocytes were transfected with small interfering RNA duplexes for PGC1β or negative control, respectively. The sequences of si-PGC1β duplexes were as follows: sense sequence, 5′-GGAGAGAGUCGCAAGAGAATT-3′ and anti-sense sequence, 5′-UUCUCUUGCGACUCUCUCCTT-3′. The sequences of negative control siRNA duplexes were as follows: sense sequence, 5′-UUCUCCGAACGUGUCACGUTT-3′ and anti-sense sequence, 5′-ACGUGACACGUUCGGAGAATT-3′. Lipofectamine^®^ RNAiMAX Transfection Reagent (Invitrogen) was used for the delivery of siRNA duplexes as described by the manufacturer.

Primary hepatocytes were isolated and cultured as in our previous study [9]. Then, the expression of *PGC1*β, *NRF1*, and *TFAM* genes, and mitochondrial DNA copies were measured following incubation of the cells with siRNA-lipid complex for 48 h. All the tests in cell culture were performed in three replicates, and each replicate was made up by pooling two wells for the tests.

### 4.6. Feeding Trial

We explored the putative regulatory role of PGC-1β in the metabolic adaptations via the diet composition as the common method.

Blunt snout bream were fed two experimental diets including a control (5% fat), and a high-fat diet (HFD, 15% fat) to apparent satiation, three times daily. Formulation and proximate composition of the experimental diets are presented in Supplemental Table S2. Each treatment was tested in three replicates (30 fish per replicate), and the trial lasted 10 weeks. At the end of the feeding trial, the liver samples were collected for analyses.

### 4.7. Gene Expression (qPCR)

Total RNA from liver was extracted using a commercial kit, as described earlier. Then, the quantity and purity of isolated RNA were determined by absorbance measurements at 260 and 280 nm, and electrophoresis was used to confirm its integrity. RNA samples were treated by RQ1 RNase-Free DNase prior to RT-PCR (Takara Co. Ltd., Japan) to avoid genomic DNA amplification. cDNA was generated from 500 ng DNase-treated RNA using ExScriptTM RT-PCR kit (Takara Co. Ltd., Japan), and the mixture consisted of 500 ng RNA, 2 µl buffer (5×), 0.5 µl dNTP mixture (10 mM each), 0.25 µl RNase inhibitor (40 U µl^−1^), 0.5 µl dT-AP primer (50 mM), 0.25 µl ExScript^TM^ RTase (200 U µl^−1^), and total volume made up to 10 µl with DEPC-treated H_2_O. The reaction conditions were as follows: 42 °C for 40 min, 90 °C for 2 min, and 4 °C thereafter.

Real-time qPCR was employed to determine gene expression based on the SYBR Green I fluorescence kit. According to the MIQE Guidelines (Bustin et al., 2011), primers were designed, and the qPCR was analyzed (Supplemental Table S1). The amplification efficiency of primers was between 90% to 110% with R^2^ = 0.999. The qPCR was performed in a Mini Option real-time detector (BIO-RAD, USA), as in our previous study [9]. The fluorescent quantitative PCR reaction solution consisted of 12.5 µl SYBR^®^ premix Ex TaqTM (2×), 0.5 µl PCR forward primer (10 µM), 0.5 µl PCR reverse primer (10 µM), 2.0 µl RT reaction (cDNA solution), and 9.5 µl dH_2_O. The reaction conditions were as follows: 95 °C for 3 min followed by 45 cycles consisting of 95 °C for 10 s and 60 °C for 20 s. Then, the fluorescent flux was recorded, and the reaction continued at 72 °C for 3 min. The dissolution rate was measured between 65 and 90 °C. Each increase of 0.2 °C was maintained for 1 s, and the fluorescent flux was recorded. All amplicons were initially separated by agarose gel electrophoresis to ensure that they were of correct size. A dissociation curve was determined during the PCR program to make sure that specific products were obtained in each run. All reactions were performed in three technical replicates. The gene expression levels were normalized towards the mean of ribosomal protein L13a (Rpl13a). The gene expression was calculated by using the comparative (2^-ΔΔCt^) method without correction for primer efficiency.

### 4.8. Mitochondrial DNA Copies and Enzymes Assay

The mitochondrial number is often determined through qPCR measurement of mitochondrial DNA (mtDNA) copies expressed relative to nuclear DNA (nDNA). The extraction of DNA was done using Qiagen DNeasy tissue ”on column” system as described by the manufacturer. The following primers were used for mtDNA (ND1): forward primer 5′ TAGCCCCTGCCTGACCACT 3′, reverse primer 5′ CTGGGATGTGGTGAATGTGTGA 3′. The primers for nDNA (beta globin) were as follows: forward primer 5′ GAATGCTCATCGTCTACCCTCA 3′ and reverse primer 5′ ATGGCTGTCATCACAGTTTTGC 3′. Gene expression was analyzed using real-time qPCR based on the SYBR Green I fluorescence kit as described in Section 4.7. The relative mtDNA copy number (the expression of ND1) was normalized towards the mean of nuclear DNA (beta globin). The gene expression was calculated by using the comparative (2^-ΔΔCt^) method [39] without correction for primer efficiency.

The activities of CS and SDH were measured using liver homogenate. Briefly, liver tissue was thawed and homogenized in nine volume ice-cold buffer (10 mM HEPES, 1 mM EDTA, 1 mM dithiothreitol, pH 7.4). The extract was later centrifuged at 850x *g* at 4 °C for 10 min. The supernatant was used to determine activity. CS activity was analyzed using a commercial kit (Nanjing JianCheng Bioengineering Institute, China) as the color change of 5,5′-dithiobis-(2-nitrobenzoic) acid (DNTB). The reaction was initiated by the addition of 0.5 mM oxaloacetate and 10 μL supernatant, and the absorbance was measured at 412 nm and 28 °C for 15 min. The negative control was used to subtract the oxaloacetate-independent acetyl CoA hydrolysis. Moreover, SDH activity was measured using a commercial kit (Nanjing JianCheng Bioengineering Institute, China) according to the reduction of 2,6- 2,6-dichlroindophenol (DPIP) at 600 nm and 28 °C for 1 min.

The activities of respiratory chain Complexes I and III were determined by commercial kits (Nanjing JianCheng Bioengineering Institute, China). All assays were performed at 28 °C in a final volume of 1 mL with 100 μL mitochondrial faction (about 10 μg protein) using a UV spectrophotometer. First, the isolation of liver mitochondria was performed in the buffer (containing 100 mM Tris-HCl, 100 mM mannitol, 300 mM sucrose, and 1 mM EDTA, pH 7.4), according to the instructions of the commercial kit (Nanjing JianCheng Bioengineering Institute, China). Liver tissue (0.1 to 0.2 g) was excised, washed in cold phosphate-buffered saline, and then homogenized in 10 volumes of cold medium. The homogenate was centrifuged twice at 800x *g* for 10 min at 4 °C. The superficial lipid layer was removed, and the remaining supernatant was centrifuged at 15,000x *g* for 15 min at 4 °C. The supernatant of the 15,000x *g* spin was considered to be the cytoplasmic fraction, and the residual pellet was the mitochondrial fraction. The pellet obtained from the second spin was washed three times in medium and was resuspended in storage buffer (containing 20 mM MOPS, 110 mM KCl, 10 mM MgCl_2_, 10 mM sodium succinate, and 1 mM EGTA, pH 7.4).

Complex I was the NADH-CoQ reductase and its activity was measured by following the decrease in absorbance due to the oxidation of NADH at 340 nm, using commercial kits (Nanjing JianCheng Bioengineering Institute, China). According to the kit instructions, the specific activity of Complex I was calculated due to the enzyme activity incubated without and with rotenone. Complex III was the uniquinol cytochrome c oxidoreductase and its activity was measured by monitoring the reduction of cytochrome c at 550 nm. Reduced ubiquinone was used as the substrate during the assay according to the commercial kits (Nanjing JianCheng Bioengineering Institute, China).

### 4.9. Statistical Analysis

Data were analyzed by SPSS 16.0, and Student’s t-test was used to compare differences between the two groups. The level of significance was set at *P* < 0.05. All data are presented as means ± standard error of the mean (SEM).

## Figures and Tables

**Table 1:** The putative phosphorylation sites of peroxisome proliferator-activated receptor-gamma coactivator-1β (PGC-1β) proteins in fish and mammals.

Phosphorylation Sites	*Homo Sapiens*	*Danio Rerio*	*Megalobrama amblycephala*
190 S			HKDGSVHRH
229 T			PVRPTGRLC
376 T		PASQTRPRM	.
391 S	HSKASWAEF		
603 S		QKSSSYKPS	QKTNSHKAS
657 T	QSDPTFGKK		
716 S		KADLSTKSS	KAELTTKSS
762 T		SKGGTQRAY	SKGESQRAY
931 S	RQLCSRSRS		
997 T		SVTQTMLRK	SFTQSMLRK

**Table 2:** Mitochondrial bioenergetics in liver of blunt snout bream (*M. amblycephala*) fed with control (5% fat) or high-fat diet (HFD, 15% fat) for 10 weeks.

	Control	High-Fat
CS (U/g prot)	14.99 ± 1.46	8.53 ± 0.68 *
SDH (U/mg prot)	3.69 ± 0.21	3.34 ± 0.31
Complex I (nmolNADH/min/mgprot)	1.42 ± 0.27	0.60 ± 0.03 *
Complex III (nmolCytC/min/mgprot)	0.21 ± 0.07	0.15 ± 0.01

All data are presented as means ± standard error of the mean (SEM, n = 6). The asterisk indicates significant difference (*P* < 0.05, Student’s t-test). SDH, succinate dehydrogenase; CS, citrate synthase. Complex, mitochondrial respiratory chain complexes.

## Supplementary Materials

The following are available online at https://www.mdpi.com/1422-0067/21/6/1935/s1, **Table S1** The primers used for cloning and expression analysis. Table S2 Formulation and proximate composition of the experimental diets. **Table S3.** Likelihood values and parameter estimates of computing position selection site by site-specific model and branch-site model for the PGC-1β family members. **Figure S1.** The nucleotide sequence of the PGC-1β cDNA in blunt snout bream (*M. amblycephala*), and the deduced amino acid sequence. **Figure S2** Multiple sequence alignment analysis of PGC-1β family proteins in different animals. The black box showed the conserved motif between different animals; the start showed the conserved sites between different animals. **Figure S3 (Continued with S2)** Multiple sequence alignment analysis of PGC-1β family proteins in different animals. The black box showed the conserved motif between different animals; the start showed the conserved sites between different animals.
