# Supplementary material for: Molecular Characterization of PGC-1β (PPAR Gamma Coactivator 1β) and its Roles in Mitochondrial Biogenesis in Blunt Snout Bream (Megalobrama amblycephala)
Source: Int J Mol Sci. 2020 Mar 12;21(6):1935. doi: 10.3390/ijms21061935 (PMC7139572; doi:10.3390/ijms21061935)
Supplement: Supplementary file 1 [file ijms-21-01935-s001.pdf]

Table S1 The primers used for cloning and expression analysis.

Primers	Sequence （5’—3’）	Use
Primers used for RACE		
PGC-1β5-R	TATCGGCAGGCAGTAC	Synthesis of the first-strand cDNA for 5’RACE
PGC-1β5-R1	TTGATGAGTTCCACTACCGA	Used with Oligo(dT)16AP for first PCR of 5’RACE
PGC-1β5-R2	AGAACTGAGGCTTCGCTG	Used with AP for nested PCR of 5’RACE
PGC-1β3-F1	AGTGGTCATCAAAAACGAGGAGCG	Used with AP for first PCR of 3’RACE
PGC-1β3-F2	GAACGGCCAGACCTCACGGCACAG	Used with RACE3-R for nested PCR of 3’RACE
Primers for real time PCR		
PGC-1β-F	CTCTAAGGGTGAATCGCAACG	Real Time PCR
PGC-1β-R	TCCTCCGCCACTTCCACAT	
NRF1-F	CTCTACGCCTTTGAGGACCAG	Real Time PCR
NRF1-R	CCAGTGCCAACCTGTATGAGC	
TFAM-F	CTTTGGTATCCAGGGAGCAGT	Real Time PCR
TFAM-R	GTTGAATCGCATCCAGTCGT	
Rpl13a -F	TCTGGAGGACTGTAAGAGGTATGC	Real Time PCR
Rpl13a -R	AGACGCACAATCTTGAGAGCAG	

Rpl13a : reference gene

Table S2 Formulation and proximate composition of the experimental diets.

Ingredients (%)	Diets	
	Control	High-Fat
Fish meal	15.0	15.0
Casein	15.0	15.0
Soybean meal	20.0	20.0
Corn starch	25.0	25.0
α-starch	5.00	5.00
Fish oil	1.90	6.90
Soybean oil	1.90	6.90
Cellulose	10.4	0.40
Calcium biphosphate	1.80	1.80
Premix [a]	1.00	1.00
Carboxymethyl cellulose	3.00	3.00
Proximate composition (%)		
Moisture	9.60	9.70
Crude protein	31.4	31.1
Crude lipid	4.80	14.6
Crude fiber	13.5	4.10
Ash	8.10	8.00
Nitrogen-free extract [c]	32.6	32.5
Energy [d] (KJ/g diet)	14.9	18.7

[a] Premix supplied the following minerals (g/kg) and vitamins (IU or mg/kg): $CuSO_4 \cdot 5H_2O$, 2.0g; $FeSO_4 \cdot 7H_2O$, 25g; $ZnSO_4 \cdot 7H_2O$, 22g; $MnSO_4 \cdot 4H_2O$, 7g; Na_2SeO_3, 0.04g; KI, 0.026g; $CoCl_2 \cdot 6H_2O$, 0.1g; Vitamin A, 900000IU; Vitamin D, 200000IU; Vitamin E, 4500mg; Vitamin K_3 , 220mg; Vitamin B_1, 320mg; Vitamin B_2, 1090mg; Niacin, 2800mg; Vitamin B_5, 2000mg; Vitamin B_6, 500mg; Vitamin B_{12}, 1.6mg; Vitamin C, 5000mg; Pantothenate, 1000mg; Folic acid, 165mg; Choline, 60000 mg.

[b] Carbohydrate (nitrogen-free extract) was calculate by difference (1000-moisture - crude protein - crude lipid – ash - crude fiber).

[c] Energy (KJ/g diet)=(%crude protein×23.6)+(%crude lipids×39.5)+ (%carbohydrates×17.3).

Table S3. Likelihood values and parameter estimates of computing position selection site by site-specific model and branch-site model for the PGC-1β family members.

Model	lnL	Parameter	Positive selection site	2ΔL	LRT
M0 (one rate)	-35183.770970	ω = 0.19324	None	0	*P* > 0.95
M1a (neutral)	-34720.522072	ω0 = 0.14008, p0 = 0.76496<br>ω1 = 1.00000, p1 = 0.23504	Not allowed		
M2a (selection)	-34720.522072	ω0 = 0.14008, p0 = 0.76496<br>ω1 = 1.00000, p1 = 0.18922<br>ω2 = 1.00000, p2 = 0.04582	Not found		
M7 (beta)	-34399.211810	p = 0.73324, q = 2.30068	Not allowed		
M8 (beta & ω)	-34396.298907	p0 = 0.96690, p = 0.79577,<br>q = 2.81250, p1= 0.03310<br>ω = 1.00000	296 E, 396 P, 402 N, 403 S, 407 P, 412 S, 673 S, 793 S, 898 A	5.8258<br>(M7 vs. M8)	*P* < 0.05
Branch-site model	-34670.949715	Background:<br>ω0 = 0.13973, ω1 = 1.00000 ω2a = 0.13973, ω2b = 1.00000<br>foreground:<br>ω0 =0.13973, ω1 = 1.00000, ω2a = 1.00000, ω2b = 999.000	705 C*, 1049 C*, 1072 Q*, 1132 S*		

#: * means that P < 0.05 by LRT test of BEB analysis.

```
1    CGCGCGGTGGGGGTGCGGGAGGCTGCGGGGTGAGTGGGGGGGGTCGATTCCGCAGCTCCGCGGACGTGGGTTGGTGGGATTCTCTAGAAGATGGCGGACTGCGCTTCACTGTTAGATGAG
1                                                                                                  M  A  D  C  A  S  L  L  D  E
121  GAGCTCTCTTCATTCGTCTTCAATTACTTGACTGAAAACTCCGGAAGCCAGTATGGGGAAGAGGAGGTCTGCTCAGACCGCCTGGATGCTGACTTCCCTGACTTTGACCTCTCGCAGCTG
11    E  L  S  S  F  V  F  N  Y  L  T  E  N  S  G  S  Q  Y  G  E  E  E  V  C  S  D  R  L  D  A  D  F  P  D  F  D  L  S  Q  L
241  GACGCCAGTGATTTTGACTCAGTGAACTGCCTCAGTGAGCTGCATTGGTGCAACGAGCACTCAGATCAATCTCCTGCATCCATACAGTACAGTGCTGGAGACCCAGAGCTATTTGAGGAA
51    D  A  S  D  F  D  S  V  N  C  L  S  E  L  H  W  C  N  E  H  S  D  Q  S  P  A  S  I  Q  Y  S  A  G  D  P  E  L  F  E  E
361  GAAAATGCGGCACTGCTCGCTGCCCTCACAGACAGCCTGGACGGCATTGTTGAGGACGGAGTCGGGGGTCTGTCTGTGTTCCCTTCCCTAGGGGACGACCCTGAGGAGGGCGAGGAGGAG
91    E  N  A  A  L  L  A  A  L  T  D  S  L  D  G  I  V  E  D  G  V  G  G  L  S  V  F  P  S  L  G  D  D  P  E  E  G  E  E  E
481  GAAGATGACCTCCCTTTGGACAGTGAGCCTTTCCCTGGCTCACTGAGCCCAGAGACAGAAGAACCATCTCTACTCAAAAAGCTCCTGTTGTCGCCCCCTAATGTGCCTGCGGGCCTCGAG
131   E  D  D  L  P  L  D  S  E  P  F  P  G  S  L  S  P  E  T  E  E  P  S  L  L  K  K  L  L  L  S  P  P  N  V  P  A  G  L  E
601  TCGCACAAAGACGGCAGCGTGCATCGCCATAGCAGCAGGAACCAGCACGTCAAGCCCGTGAGGCCTGTGCTGAAGAAGGACAAATCAAGCACACAGGAGCGTAAGCCACGACCAGTGAGG
171   S  H  K  D  G  S  V  H  R  H  S  S  R  N  Q  H  V  K  P  V  R  P  V  L  K  K  D  K  S  S  T  Q  E  R  K  P  R  P  V  R
721  CCCACCGGTCGTCTTTGCACTGAACTCCACCGCCACCTCACCACCGCACGGGAGGCTGAGGACACGCCTTCCGCTGACACGGAGGAAGAGGAAGAGGAGGATGAAGAGGACAGTGACTCA
211   P  T  G  R  L  C  T  E  L  H  R  H  L  T  T  A  R  E  A  E  D  T  P  S  A  D  T  E  E  E  E  E  E  D  E  E  D  S  D  S
841  GAGGAAGAGGAGGAGGAGGAGTCTTCCAGCAGTGAGAGTGAGAGCATGGTGTGCGTTGAGCCAGCGAAGCCTCAGTTCTCCTCAGAGAAGGAACTGCACTCGGTAGTGGAACTCATCAAA
251   E  E  E  E  E  E  E  S  S  S  S  E  S  E  S  M  V  C  V  E  P  A  K  P  Q  F  S  S  E  K  E  L  H  S  V  V  E  L  I  K
961  TACATGCACACGTACTGCCTGCCGATACGCAAACAGGCCAGCTGGGATCGCAAAGAGCGTGAGGCTTTGGCGCGAAAGACCAAGCCTGAGAACCCGCAGGTCCCCCGTGGCCTTCCTCAG
291   Y  M  H  T  Y  C  L  P  I  R  K  Q  A  S  W  D  R  K  E  R  E  A  L  A  R  K  T  K  P  E  N  P  Q  V  P  R  G  L  P  Q
1081 AACTCTCAGAAACCGCCCCCCCAGACCAGCAGTGGGACCAGGCCAAGGGTGCCCTTCACTCGCCGCAGGGAAATCAAAGCCCACTCCCTGCTCAAGGAGCTGTTAGAGGCAGTCAGTTCC
331   N  S  Q  K  P  P  P  Q  T  S  S  G  T  R  P  R  V  P  F  T  R  R  R  E  I  K  A  H  S  L  L  K  E  L  L  E  A  V  S  S
1201 TTTGACGTAAGCAAGCCTTACAGAATGCACAGCCCCCCTTATATCCACTGCAGAGGGGCAGTGACTCGGCCTGGCGCTGACGTTTCTTCCTCCCCTGCACGTCAGCCCAAAGCTGAAGCT
371   F  D  V  S  K  P  Y  R  M  H  S  P  P  Y  I  H  C  R  G  A  V  T  R  P  G  A  D  V  S  S  S  P  A  R  Q  P  K  A  E  A
1321 AAAGATTCAGACTGCGAGAGCTCGCAAAAGGCCGCAAAGCGCCCCAAAAGCCCCGAGCCAGAGGAAGGCTCCTTTTCAGTCAGGCGTTCCCGCCGTCTAGCCTCCTTCCCCAGCCGATTC
411   K  D  S  D  C  E  S  S  Q  K  A  A  K  R  P  K  S  P  E  P  E  E  G  S  F  S  V  R  R  S  R  R  L  A  S  F  P  S  R  F
1441 GCTAAGAAGGTCCGTGAGAACTGTGGGCGGTTGGAGCCCATTGCTGGGCAATCCAGGGAAGAGGAGAACGTGGTCAAACATCCTCCCACTGAGCCTCCTTCTGATAGCAGCCAGAAGACC
451   A  K  K  V  R  E  N  C  G  R  L  E  P  I  A  G  Q  S  R  E  E  E  N  V  V  K  H  P  P  T  E  P  P  S  D  S  S  Q  K  T
1561 AACTCCCACAAGGCTTCTGAAGCCCATAACCCATGCTGCAGGGACGAGAAACGCTCCTGCCTCTGTCTGCCGCTGGCGTCCAAATCCAATGGAGACACGCAGTATGCCAACAAGCCCTTT
491   N  S  H  K  A  S  E  A  H  N  P  C  C  R  D  E  K  R  S  C  L  C  L  P  L  A  S  K  S  N  G  D  T  Q  Y  A  N  K  P  F
1681 GAGCAGACTCTCTGTGTGGAATTGTGTGGCACAGCAGGCCTGACTCCCCCCACCACACCCCCTCATAAACCAGTGGAGGATGAGCTCTTCAAACCAGACGGAAAAGCTGAGCTCACCACC
531   E  Q  T  L  C  V  E  L  C  G  T  A  G  L  T  P  P  T  T  P  P  H  K  P  V  E  D  E  L  F  K  P  D  G  K  A  E  L  T  T
1801 AAGAGCTCGTGCCTGGCGCGGGCGCACATACGCAAGCTCCCAGAGCAGACAGAACTGTACGCCCAGCTGCGCAGGATGGGCCAAACGGGCGACACGGACTCTAAGGGTGAATCGCAACGT
571   K  S  S  C  L  A  R  A  H  I  R  K  L  P  E  Q  T  E  L  Y  A  Q  L  R  R  M  G  Q  T  G  D  T  D  S  K  G  E  S  Q  R
1921 GCATACGGGGACCACGATTACTGCCTATTGGGACTGGGAGAGAGTCGCAAGAGAACGGCAGCGGCGCTCGCCTCTCAGTGCCATGTGGAAGTGGCGGAGGAGGATGAGATGGATGTGAGG
611   A  Y  G  D  H  D  Y  C  L  L  G  L  G  E  S  R  K  R  T  A  A  A  L  A  S  Q  C  H  V  E  V  A  E  E  D  E  M  D  V  R
2041 AACGGGGAGATTGAGGGACAGGAGGAGAGGCTCCTAACGAAGGTCCCCGATTACCAGCAAACGACTGAGGCCGACTCGGCCACTGCGTTGACCACGCCCAGTCCCGAGCAGACCAGTGAA
651   N  G  E  I  E  G  Q  E  E  R  L  L  T  K  V  P  D  Y  Q  Q  T  T  E  A  D  S  A  T  A  L  T  T  P  S  P  E  Q  T  S  E
2161 CCCATACCTGTCCGTTCTCCCAGCCCAGAGCTGGACGCCCAGTCCCCAGTTTCCTGTTCACCCCCCTCACCCAGCTGCAAACTCTCCTTCAGCGATGAGAGCTCTGAGACATGCCACGAA
691   P  I  P  V  R  S  P  S  P  E  L  D  A  Q  S  P  V  S  C  S  P  P  S  P  S  C  K  L  S  F  S  D  E  S  S  E  T  C  H  E
2281 GCAACTGAAAAAAGGAGCAAGACAAAGTGTGGACAAGAGAATGATGTGAACAAGTGCCAAGTGATCTACATTCACAACCTTCCGAACAGCTTCACTCAGTCCATGCTGCGTAAGCGCTTT
731   A  T  E  K  R  S  K  T  K  C  G  Q  E  N  D  V  N  K  C  Q  V  I  Y  I  H  N  L  P  N  S  F  T  Q  S  M  L  R  K  R  F
2401 GAGGCCTTCGGCCGTCCAGAAGACTGTAAAGTGGTCATCAAAAACGAGGAGCGCTGTGGAGTGATCACACTGAGGCCCACTCAGAACGGCCAGACCTCACGGCACAGGTGGGACTCGCTT
771   E  A  F  G  R  P  E  D  C  K  V  V  I  K  N  E  E  R  C  G  V  I  T  L  R  P  T  Q  N  G  Q  T  S  R  H  R  W  D  S  L
2521 GGTCCGAGCGGAGGGAACGGCAGCCGGCGGTTCGGCAGGAGGCGTTACATAGATCTGGATGAGGCGGGTCCAGGTCCGGTGAAGAGCAAGTACGATGCACTGGACTTTGACGCTCTGCTG
811   G  P  S  G  G  N  G  S  R  R  F  G  R  R  R  Y  I  D  L  D  E  A  G  P  G  P  V  K  S  K  Y  D  A  L  D  F  D  A  L  L
2641 AAAGAGGCCCAGAGAAGCCTGCATCGCTGACACGCCTTACCACGCCTCCAGCGACACGCCCTGTCCGAGACTCTCCTCAGCACCTCAACCAAGGCACTTCTTCATGTTTACATTTTGAAC
851   K  E  A  Q  R  S  L  H  R  *
2761 ATTGGAATAAAAAATAGACCTTGTCACTTCTAACTGAGGATCGGAGGACATTGGGGCGGGAGGATTACAAACGAACAGAAACAGCTCTGACACACTGGACACAAGTGCTGCTTCTCGTGT
2881 GTGTGTTTGATGCCATTCATGACGTTGCGAGCGGCTCGGGCTTCGTAGCAAACCGCACTCTTTTCCATTGGTTGTTGTGACGTGCGTGTGCTTTTTTTTTGTTTTTTTATTTGTTTCCCT
3001 TGTCTTCTTTTCATAGACCTGCAAGGTGCTTTCACCATTTTGTCGTACAAATATATAAACAATAACTGCTTTGTATTAGAAAAAAAAAAAAAAAAAAAAAAAAAAAAAAAA
```

Figure S1. The nucleotide sequence of the PGC-1β cDNA in blunt snout bream (*M. amblycephala*), and the deduced amino acid sequence.

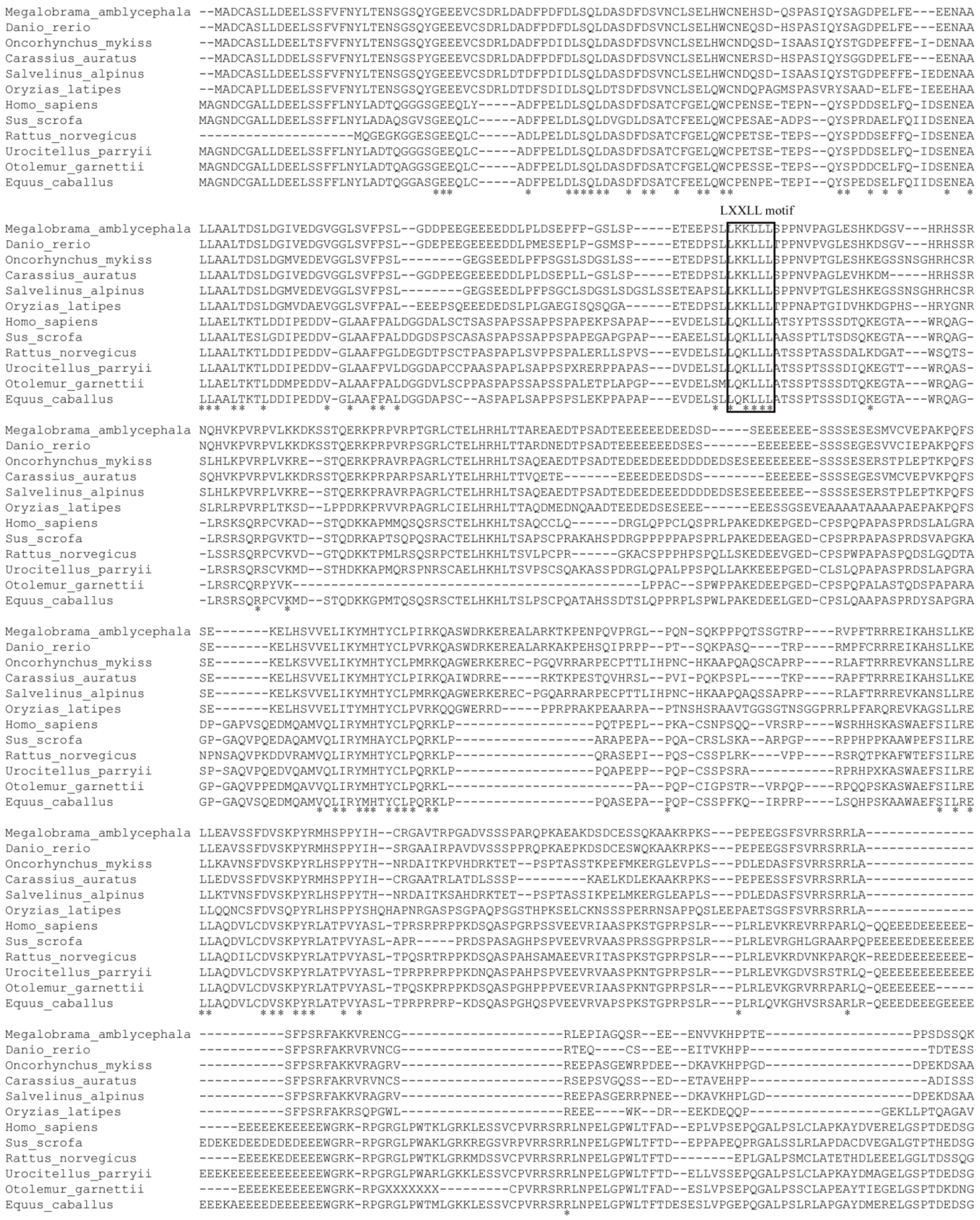

Figure S2 Multiple sequence alignment analysis of PGC-1β family proteins in different animals. The black box showed the conserved motif between different animals; the start showed the conserved sites between different animals.

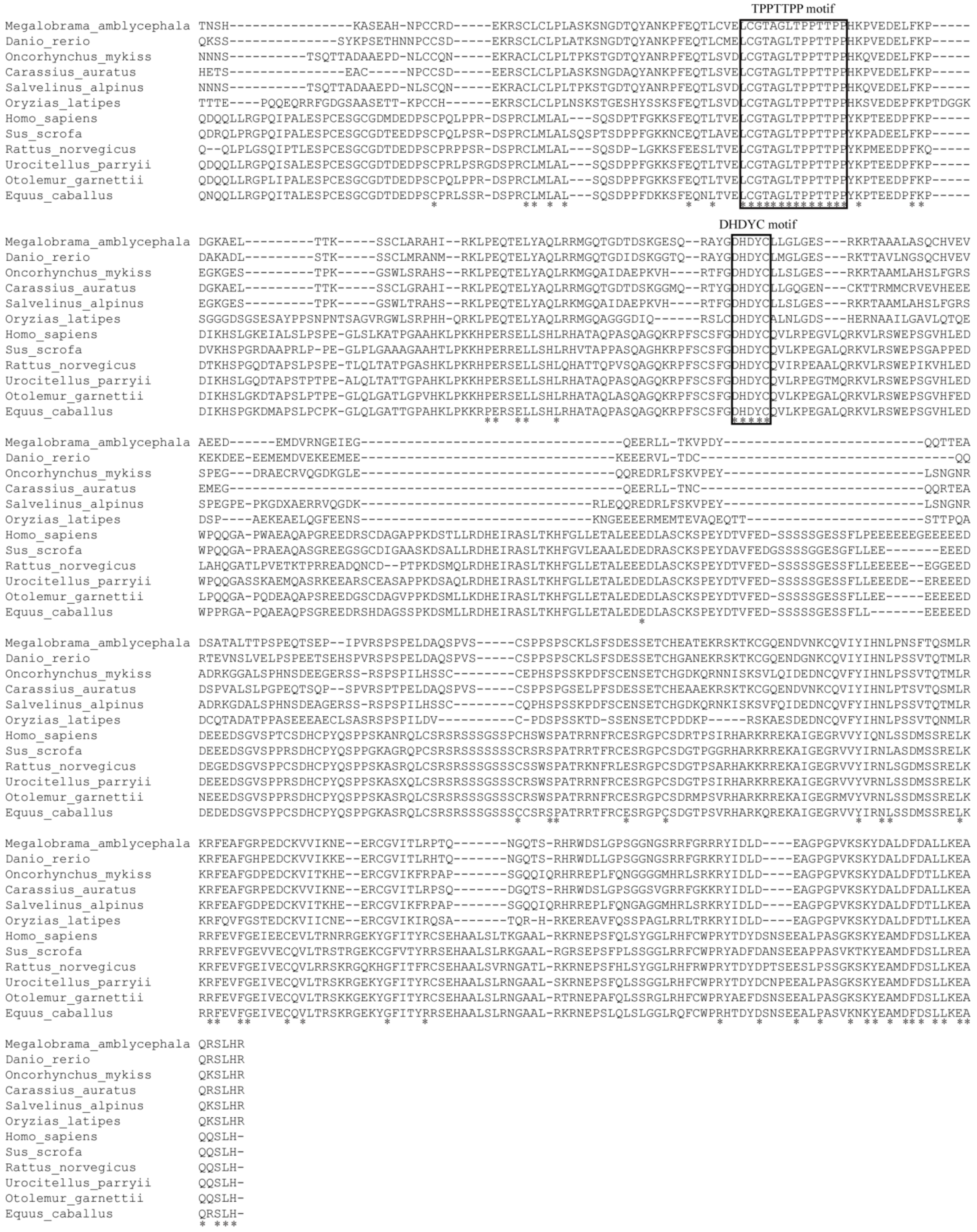

Figure S3 (Continued with S2) Multiple sequence alignment analysis of PGC-1β family proteins in different animals. The black box showed the conserved motif between different animals; the start showed the conserved sites between different animals.
